# Applying advanced technologies to improve clinical trials: a systematic mapping study

**DOI:** 10.1007/s11192-020-03774-1

**Published:** 2020-11-21

**Authors:** Esther Nanzayi Ngayua, Jianjia He, Kwabena Agyei-Boahene

**Affiliations:** 1 School of Business, University of Shanghai for Science and Technology, Shanghai, 200093 China; 2 School of Medicine, Tongji University, Shanghai, 200092 China

**Keywords:** Artificial intelligence, Machine learning, Deep learning, Internet of things, Clinical trials

## Abstract

The increasing demand for new therapies and other clinical interventions has made researchers conduct many clinical trials. The high level of evidence generated by clinical trials makes them the main approach to evaluating new clinical interventions. The increasing amounts of data to be considered in the planning and conducting of clinical trials has led to higher costs and increased timelines of clinical trials, with low productivity. Advanced technologies including artificial intelligence, machine learning, deep learning, and the internet of things offer an opportunity to improve the efficiency and productivity of clinical trials at various stages. Although researchers have done some tangible work regarding the application of advanced technologies in clinical trials, the studies are yet to be mapped to give a general picture of the current state of research. This systematic mapping study was conducted to identify and analyze studies published on the role of advanced technologies in clinical trials. A search restricted to the period between 2010 and 2020 yielded a total of 443 articles. The analysis revealed a trend of increasing research interests in the area over the years. Recruitment and eligibility aspects were the main focus of the studies. The main research types were validation and evaluation studies. Most studies contributed methods and theories, hence there exists a gap for architecture, process, and metric contributions. In the future, more empirical studies are expected given the increasing interest to implement the AI, ML, DL, and IoT in clinical trials.

## Introduction

The pharmaceutical landscape is rapidly evolving in a manner that is causing an increase in costs and timelines for conducting clinical trials. Randomized clinical trials (RCTs) are the epitome of providing the highest level of evidence for the causal relationship between a clinical intervention and the target outcomes, hence they are commonly applied in the innovation of medical solutions (Mayo et al. 2017; Sen et al. 2017). The increasing costs of clinical trials happening amidst a declining global output of medical innovations is a cause for alarm. The last decade is characterized by several failures in drug launches happening at a rate that could be the highest ever encountered (Lodha 2016). About one in every five clinical trials do not complete enrollment of participants due to reasons such as complexities of applying the stringent eligibility criteria and rigid inclusion criteria (Beck et al. 2020). Thus, there is a need to have a system that utilizes the data generated in the course of planning and executing clinical trials to increase the visibility of problems, possibilities, and progress. Such a system can enhance adaptability to the constantly dynamic regulatory requirements and clinical practices of RCTs.

Subject enrolment forecasting is one of the main obstacles that derail the success of clinical trials. Sites, patient populations, and sponsors continue to be vexed by difficulties in the enrollment of representative samples for clinical trials (Calaprice-Whitty et al. 2020). For example, selecting and matching subjects for an oncology clinical trial can be difficult given the patients’ genetic makeup and tumor heterogeneity (Taglang and Jackson 2016). For a trial to be successful, it has to attain an adequate enrolment of properly-matched research participants (Lodha 2016). Challenges in subject enrolment and matching cause extension of enrolment deadlines, delay the submission of the trial protocols for regulatory approvals, and subsequently cause a deferment of the product launch beyond the initially planned dates. Selection bias can lead to results that are not generalizable because populations that were underrepresented may not respond well to the intervention (Mayo et al. 2017; Sen et al. 2017). The challenges can also lead to under- or over-enrollment, hence increasing the overall cost of the clinical trial (Lodha 2016).

The availability of data from potential subjects, including medical history and details of their genomic variations presents an opportunity for an advanced selection and matching of RCT participants even from a previously unknown population (Mayo et al. 2017; Taglang and Jackson 2016). Big data clinical trials can provide researchers with a large sample of data to enhance RCTs. Machine learning and advanced data analytics can be applied in the prospective planning of clinical trials to ensure the validity of the RCTs (Mehta et al. 2019). Developing clinic-molecular data analytics can help in the understanding of the biology and clinical status of participants in a clinical trial (Taglang and Jackson 2016), thus facilitating subject matching. Artificial intelligence (AI)-guided clinical trials may allow the investigator to appropriately select and randomize participants using AI-assisted randomization techniques (Krittanawong et al. 2019). AI-powered technologies such as Mndel.ai can be applied in overcoming the biases and challenges encountered when prescreening populations for inclusion in a clinical trial (Calaprice-Whitty et al. 2020).

The innovative trials applying sophisticated models that capture the breadth and depth of the available data can improve multiple aspects of clinical trials to extents that the traditional statistical methods could not (Krittanawong et al. 2019; Shah et al. 2019). Applications for clinical trial matching that use predictive analytics can be augmented with standard evaluation techniques to enhance their utility in extracting populations for clinical trials from electronic health records and previous clinical trials (Krittanawong et al. 2019; Shah et al. 2019). AI and machine learning are promising strategies for the testing of intervention algorithms (Shah et al. 2019), for example, schemes for subject matching, towards better execution of clinical trials. AI-guided clinical trial matching systems can be valuable in screening patients for trial eligibility given that evaluations of such systems have revealed that they are faster yet reliable in the identification of the appropriate subjects for clinical trials (Beck et al. 2020). Therefore, opportunities exist to apply AI and other advanced technologies in the modification of factors that are associated with the failure of clinical trials(Fogel 2018). Challenges in the recruitment of participants can be addressed by leveraging on AI tools to optimize subject matching.

Several researchers have published articles to inform the application of advanced technologies in clinical trial steps such as recruitment and eligibility screening. However, the information is scantly synthesized in a manner that would provide investigators with a quick source of credible information to inform their decisions regarding the application of AI. To improve an understanding of the value of AI, machine learning, deep learning and the internet of things in planning and conducting clinical trials, we conducted a systematic mapping study utilizing the multiple and diverse literature on the topic (Petersen et al. 2015). This paper presents the research method employed, the results of the study, and a discussion of the findings while considering limitations and future implications.

## Research method

**Fig. 1:**
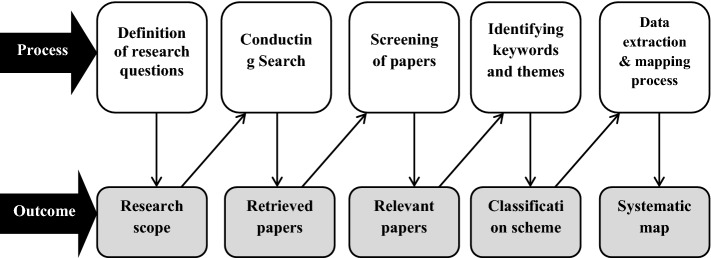
Mapping process (adapted from Peterson et al. 2008)

This part outlines the process that was followed when conducting the systematic mapping study. The method is borrowed from the mapping process described by Peterson et al. (2008, 2015). The process started by defining the research questions; identifying the search terms and doing the search; assessing the identified papers based on the inclusion and exclusion criteria; determining the major keywords and designing a scheme for their classification, extracting the data and mapping. The mapping process, which was adapted from Peterson et al. (2008, 2015), is shown in Fig. 1 below.

### Definition of research questions

This systematic mapping study aims to establish the evidence base on the application of artificial intelligence, machine learning, deep learning, and the internet of things in the planning and execution of clinical trials. The main research question of the study is:

*What is the existing evidence base regarding the use of artificial intelligence, machine learning, deep learning, and the internet of things to enhance patient matching when conducting clinical trials?*

To answer the main research question, the following objectives were set:

1. To stratify published research work regarding the use of artificial intelligence (AI), machine learning (ML), deep learning (DL), and the internet of things (IoT) in the planning and execution of clinical trials.
2. To highlight the current trends in research work and assess the value of the research efforts in on the application of AI, ML, DL, and IoT in clinical trials.
3. To identify opportunities for future research in the field of AI, ML, DL, and IoT -guided design and execution of clinical trials.

The next step of the study entailed deriving five research questions from the objectives. Responding to the questions with information gathered from research materials facilitated the synthesis of what has been happening in the area of AI, ML, DL, and IoT -informed clinical trials. The summary of the practices serves as a background from which the future trajectory of research and industrial activities can be derived. Below are the five research questions and the justification for their inclusion in the study:

- RQ1: Which types of research studies have been completed to investigate the role of AI, ML, DL, and IoT in the improvement of clinical trials?

Rationale: Answering this question will help in the identification of the types of formal studies that have been completed in the area of interest. The identified studies will be categorized according to their research approach, such that evaluation studies, validation studies, and expert opinions among others will be grouped into their respective categories to facilitate the assessment of the level and quality of evidence that is available regarding integration of AI, ML, DL, and IoT in clinical trials.

- RQ2: What forms of contributions have the studies made in the area of AI, ML, DL, and IoT-guided clinical trials?

This question will result in the clear identification of the outputs of the research efforts in the clinical trials. Responding to the question will streamline the determination of the maturity level of the research work that has been completed so far. The spectrum of maturity ranges from the less mature as provided in studies that focus on the development of theories and conceptual models, to more mature research that centers on the investigation of the related tools through implementation and evaluation studies.

- RQ3: Which aspect of clinical trial do the identified studies focus on?

Rationale: The question will be pivotal in determining the areas of clinical trial that have been substantially studied in the context of AI and the ones that have been only scantly studied. Therefore, it will be possible to highlight the aspects of the issue that have been extensively studied and identify the areas that need more research.

- RQ4: What study dimensions feature in the considered studies?

Exploring the study dimensions, which include execution, technical, research, regulation, and policy will reveal the secondary realms of research that the studies focus on besides the primary area, which is the application of AI, ML, DL, and IoT in clinical trials. Several studies have multiple study dimensions hence the need to consider all the dimensions and classify the studies under the most relevant categories to avoid omission of essential information.

- RQ5: What is the pattern of research evolution on the issue of AI, ML, DL, and IoT in clinical trials guided clinical trials?

Rationale: Answering the question will help in the distribution of the identified research over the years, thus facilitating the identification of trends in the field. Thus, clinical researchers and regulatory bodies can understand the identified trends to inform their decisions regarding the investigation or application of AI, ML, DL, and IoT in clinical trials.

### Conducting the research

We began the process of answering the five research questions by formulating the search terms that would help in the identification of the relevant research articles. Then we selected data sources and searched for the articles from them using the search terms.

#### Search criteria

The PICOC (population, intervention, comparison, outcome, and context) criteria as described by Petticrew and Roberts (2008) were applied in defining the search terms that were used in the search. For our study, the population comprises the clinical trials. The phenomenon of interest, which can also be regarded as the intervention as per the PICOC criteria, is the application of AI, ML, DL, and IoT in clinical trials. Therefore, the context as represented by the last C in PICOC is the planning and execution of clinical trials. There was no consideration of the comparison and outcome in the definition of the search terms because the study is exploratory and did not focus on any particular outcome or compare the AI, ML, DL, IoT with any other approach.

The selected keywords were grouped into sets based on the criteria. Where possible, the synonyms of the keywords were considered to ensure that the search was as exhaustive as possible. The first set, comprising of search terms related to the population, comprised of the following search terms: “clinical trial”, “randomized controlled trial”, and “trial”. The second set of search terms was intervention-related and included “artificial intelligence”, “machine learning”, “deep learning”, “internet of things”, “advanced technologies”, “big data”, “analytics”, and “data science.” The third set was context-related and comprised “planning”, “conducting”, “recruiting”, “eligibility screening,” and “patient matching”.

#### Data sources and search process

The databases searched include PubMed, Embase, and Trip Pro, which are all digital databases. Sets of searches were conducted in the databases from February 1st to March 15th using combinations of the formulated search terms. All articles published in English between January 2010 and February 2020 were considered for the systematic mapping study. The search criteria and the results obtained in every stage are outlined in Table 1.

**Table 1:** Searches in digital databases for articles published from January 2010 to February 2020

Digital database	Keywords searched	Content type	Search results
PubMed	“Clinical trial” OR “randomized controlled trial” OR “trial” OR “drug discovery” OR“vaccine testing” AND “artificial intelligence” OR “machine learning” OR “deep learning” OR “internet of things” OR “advanced technologies” OR “big data” OR “analytics” OR “data science” AND “planning” OR “conducting” OR “recruiting” OR “eligibility screening”	Research articles, expert opinions, review articles, evaluation studies, systematic reviews	92,714
Embase	“Clinical trial” OR “randomized controlled trial” OR “trial” OR “drug discovery” OR“vaccine testing” AND “artificial intelligence” OR “machine learning” OR “deep learning” OR “internet of things” OR “advanced technologies” OR “big data” OR “analytics” OR “data science” AND “planning” OR “conducting” OR “recruiting” OR “eligibility screening”	Research articles, expert opinions, evaluation studies, review articles	232,732
Trip Pro	“Clinical trial” OR “randomized controlled trial” OR “trial” OR “drug discovery” OR“vaccine testing” AND “artificial intelligence” OR “machine learning” OR “deep learning” OR “internet of things” OR “advanced technologies” OR “big data” OR “analytics” OR “data science” AND “planning” OR “conducting” OR “recruiting” OR “eligibility screening”	Clinical trials, evaluation studies, technical reports, validation studies, mini-reviews	7640

### Screening of papers

The yields of the search were first screened during the search process where filters were applied to limit the search results to articles that were published within the last ten years. Another filter excluded articles that were not available in full-text form. Intensive manual screening then followed, whereby the articles for use in the systematic mapping study were identified by assessing the titles, abstracts, and keywords of the articles that emerged from the three databases searched. Skimming through the full-text articles further helped to screen the articles, and articles that elicited doubt regarding relevance in the process of skimming were read in full to ascertain their appropriateness.

The inclusion criteria entailed the selection of peer-reviewed journal articles published in English between January 2010 and February 2020. The articles had to be explicitly mentioning the keywords or the synonyms of the keywords in the topic of this study. The keywords include artificial intelligence, machine learning, deep learning, internet of things, clinical trial, participant, recruitment, and eligibility. Therefore, articles that were only available in abstract form; published in books, editorials, and practice guidelines; authored in non-English languages; or appearing as duplicates were excluded from the study.

**Fig. 2:**
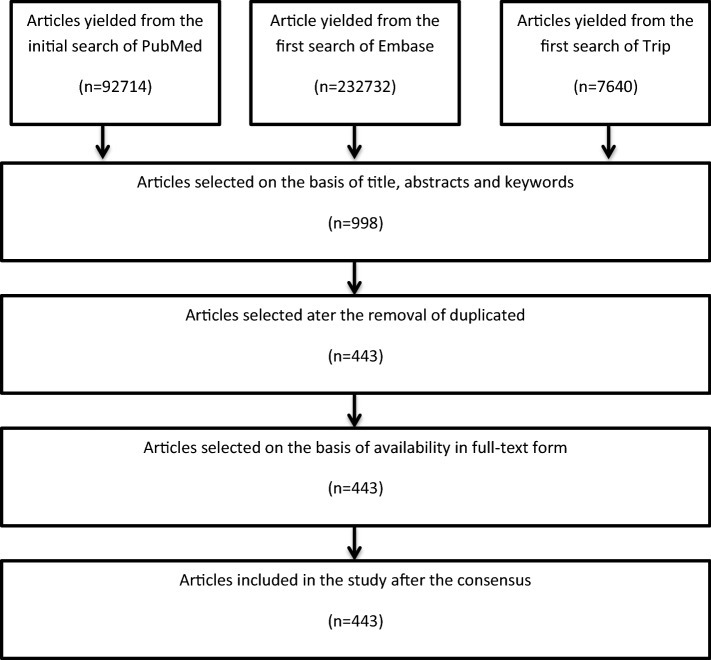
PRISMA flow diagram showing the process of selection of articles

The primary author completed the search process and applied the inclusion and exclusion criteria to select the most appropriate articles for the study. Two more authors analyzed the selected articles to verify their relevance for the study in line with the requirements of the observer triangulation method. The next stage entailed the categorization of the selected articles by the primary author. The assignment of the articles into the various categories was affirmed after consensus by the three authors. The total number of articles included for evaluation is 998. The articles’ selection process was as shown in the Preferred Reporting Items for Systematic Reviews and Meta-Analysis (PRISMA) flow diagram shown in Fig. 2.

### Designing of a classification scheme

Five different facets were designed to help in answering the five research questions and obtaining a broad view of the current status of research in the field of advanced technologies in clinical trials. The five facets were derived from two classification schemes that were designed to classify the articles obtained from the screening criteria. The fixed classification scheme produced three facets namely publication year, research type and contribution facets. The topic-specific classification scheme yielded two facets namely study focus and study dimension. The selection of the facets was guided by the proposals of Petersen et al. (2008, 2015). Below are details of each of the five facets:

The publication year facet allowed the display of the studies based on their frequency distribution across the 2010 to 2020 period. The facet made it possible to determine the trend at which studies related to the topic were done over the years. For instance, the researchers could tell which years were characterized by the publication of several research papers and which years recorded low numbers of publications related to the utilization of AI, ML, DL, and IoT in clinical trials.

The research type facet led to the categorization of the articles considered in the study based on the research approaches used. The categorization followed the format proposed and explained by Wieringa et al. (2006) and Roul and Sahoo (2017). The research types include validation, evaluation, solution proposal, philosophical, opinion, discussion, study protocols, and experience studies as shown in Table 2. The research types can give a hint on the evidence hierarchy of the included studies.

**Table 2:** Research type facet borrowed from Petersen et al. (2015) and Wieringa et al. (2006)

Category	Description	Applicable conditions
Evaluation research	A study that tests the success of a particular intervention after it is implemented; it could also mean research that examines the application of a particular intervention such as AI in a given context such as clinical trials	Empirical evaluation and in practice
Experience paper	An article that describes the journey of organizations that have gone through the process of implementing a strategy such as the AI-guided selection of research participants	Empirical evaluation
Validation research	Research that assesses proposed solutions that are not yet implemented	Empirical evaluation
Solution proposal	A study that proposes a solution that is yet to be validated to a problem, for example through a proof of concept	Novel solution
Philosophical paper	An article that presents a new dimension of viewing an existing thing by describing a new conceptual framework. Review papers that provide a detailed description of a topic also fall under this category	Conceptual framework
Study protocol	A paper that outlines the details of a study that is set to be conducted; it describes the objectives, study design, and methods of the proposed research	Conducting a study
Discussion papers	Papers that present balanced information regarding a topic so that stakeholders can take up the issue and discuss it to reach a decision. They are mainly reports of committees, conferences, or meetings	Opinion
Opinion papers	Articles that present the views of the author regarding a particular subject	Opinion
Review paper	A paper that synthesizes findings of previous research papers, systematically or otherwise, to gather evidence on a topic of interest	Systematic review Literature review

The contribution facet classified the articles based on the outcomes of the studies. It resulted in eleven contribution types as guided by the work of Shaw (2003). The categories include architecture, framework, method, metric, model, platform, process, strategy, system, theory, and tool as shown in Table 3.

**Table 3:** Contribution facet adapted from Shaw (2003)

Category	Description of the category
Metric	Presents a quantitative measure for estimating the degree of possession of a certain characteristic by a component of a system, the system itself, or the processes that take place in a system
System	Describes the components and elements that form what is required to complete a particular activity
Architecture	Outlines how a system is organized in terms of the constituent components and the interactions among them
Process	Describes the set of activities that ought to be completed to solve a defined problem
Method	Proposed a procedure for applying a specified intervention in the solution of a problem
Framework	Suggests a structure for establishing a solution for a specific issue
Model	Formulates a mathematical or conceptual model for tackling a specified issue
Tool	Develops an item that is useful n the implementation or maintenance of a proposed solution
Strategy	Recommends a plan for accomplishing a long-term Goa
Theory	Explains the basis of a study by outlining the involved principles
Platform	Offers the hardware or software to host the proposed solution

The study focus facet provides details of the clinical trial that each of the selected studies focuses on. The classification in this focus was facilitated by the identification of the context of the individual research studies. Given that the systematic map focuses on all the stages of a clinical trial, the contexts of the studies were derived from their planning to completion of the clinical trials. Thus, Table 4 was prepared with summaries of categories of the area of clinical trials.

**Table 4:** Study focus (the aspect of the clinical trial)

Category	Description
Recruitment	Studies that focus on the planning and preparation step of the recruitment process
Eligibility	Studies that center on ensuring that clinical trials get participants who meet the specified inclusion criteria
Patient matching	Studies that focus on improving the matching clinical trial subjects with the help of AI
Ethics	Studies that concentrate on ensuring that clinical trials adhere to the set ethical standards such as consenting
Interventions	Studies that are about the administration or monitoring of the effect of an intervention in a clinical trial
Overview	Studies that are not specific on a particular aspect of a clinical trial but address the trials in general

The study dimension facet takes care of the fact that some of the included studies did not focus on a single specific area of clinical trials. It is about categorizing the secondary areas that some of the articles centered around. The classification is as shown in Table 5.

**Table 5:** Study Dimension

Category	Description
Execution	Studies that entail implementing interventions to enhance the execution of clinical trials
Technical	Studies that feature the development of mathematical or software models that can be applied in the improvement of clinical trials
Research	Studies whose focus is on the research aspects of optimizing the trial processes
Policy	Studies that target to drive policy changes in the processes of clinical trials
Regulation	Studies that aim to change some regulatory aspects of clinical trials

### Data extraction and mapping of results

#### Mapping process

Data extraction and the categorization of the selected research articles followed the design of the classification scheme. The articles were sorted into the various categories in a spreadsheet, which facilitated further data extraction and synthesis to answer the research questions. The assignment of the studies into the various categories was based on the assessment outcomes of looking at their titles, abstracts, introduction, and conclusion. When it became a challenge to use the above features to classify the studies, the articles were studied in greater detail to facilitate their classification. After the categorization process was completed, the frequency of publications was calculated using the final results. The frequencies and the combinations of the research questions aided the establishment of the systematic map that gave an overview of the application of AI, ML, DL, and IoT in the improvement of clinical trials. The rigor of the systematic map was enhanced by involving the three researchers in its design through the observer triangulation method. The developed map shows the distribution of the currently available relevant research studies. It can help in the identification of areas that require further research.

A map of the various categories of the articles was established using a bubble plot. The bubbles are of different sizes depending on the intersection of categories in the x-y scatterplot created. The diameter of each of the bubbles corresponds to the frequency of publications completed under the intersecting pair of categories. Bubble plots for the five facets show the trend of research in the area explored. Compared to other forms of data presentation, bubble plots were preferred because they provide an effective visualization of the various facets. A quick view of the systematic map can provide the reader with a snapshot of the current status of research in the application of AI, ML, DL, and IoT in clinical trials.

#### Mapping results

One of the bubble plots contains the study focus and research type facets alongside the years of publication. The study dimension and contribution of studies against years of publication were mapped in another bubble plot to demonstrate the development of research over the years during which the studies were conducted. With the help of the bubble plots, it was possible to evaluate the results of the studies and extract answers for the research questions.

## Analysis of mapping results

The search for articles regarding the application of AI, ML, DL, and IoT in clinical trials with publication dates between January 2010 and February 2020 yielded a total of 443 articles. The five research questions provided the context for the analysis of the mapping results. Answering the research questions provided information regarding the different aspects of advanced technologies in clinical trials, which resulted in a comprehensive presentation of the research area. The evidence gathered in the mapping process is discussed in detail under the subsections of the research questions.

### Research type (RQ1)

The studies identified through the search process were categorized according to the research type. After computing the counts and percentages of the studies under each research type, it was evident that most studies used the validation approach, constituting 28% of all the articles as shown in Table 6 in the appendix. The studies examined the applicability of AI, ML, DL, and IoT in various aspects of conducting clinical trials. The second and third most common research approaches include evaluation and solution proposal at 21% and 16.5% respectively. The evaluation studies measured the effect of utilizing the various information technologies in the planning and execution of clinical trials. The least utilized approaches include opinions (0.9%), study protocols (2%), and experience (4.5%). The domination of validation, evaluation and proposed solution studies, which cumulatively comprise two-thirds of the total number of studies, demonstrates the commitment by scientists to integrate information technologies in clinical trials.

### Contributions (RQ2)

The categorization of the studies in consideration of their contributions is as shown in Fig. 4. Most of the studies described theories and methods (22% and 19% respectively) as shown in Table 7 in the “Appendix”. The studies that contributed theories were mainly discussion papers (34), review articles (25), philosophical papers (16), and solution proposals (13). Their content constituted factors affecting the application of AI, ML, DL and IoT in clinical trials; the involved concepts and information; and the perceptions and considerations in the implementation. The articles that focused on methods mostly validated (33), evaluated (18) or proposed (14) interventions with aspects of AI, ML, DL and IoT for clinical trials.

Other key contributions are strategy (14%), tools (10%), platforms (10%), and system (9%). The least contributions include architecture (1%), framework (2%), process (2%), metrics (4%), and models (7%).

The applications of the specific advanced technologies is diverse across the articles. The frequency of utilization of the technologies is as shown in Fig. 5.

### Study focus (RQ3)

**Fig. 3:**
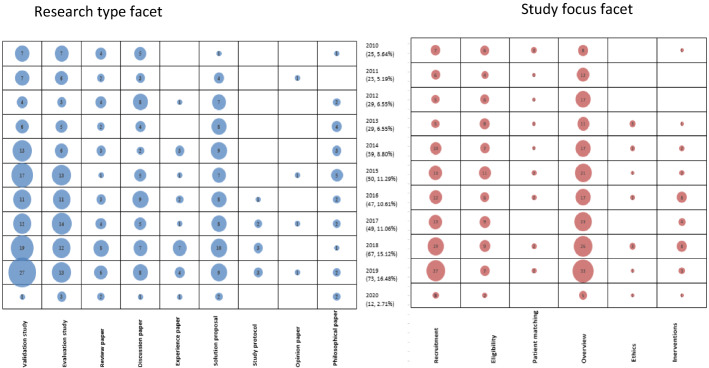
Systematic map: Bubble plot showing the trend of research according to research type and study focus, bubble size represents the number of articles

Mapping results indicate that most of the publications were about the application of the AI, ML, DL, and IoTs in the entire clinical trial process, as shown by the 43% studies under the overview study focus (Fig. 3). Regarding the focus on the individual components, recruitment and eligibility were the most targeted, at 27% and 17% respectively. Patient matching, which can also be considered part of the recruitment aspect of clinical trials, was specifically addressed by 3% of the studies. The other autonomous aspects that researchers focused on are interventions and ethics; 7% and 3% of the articles specifically addressed them respectively. The high attention given to recruitment, eligibility, and patient matching, which comprise cumula

tively 47% of the articles, shows that scientists may have noted a great potential for AI, ML, DL, and IoT in improving the processes of engaging patients to participate in clinical trials.

### Study dimension (RQ4)

This mapping exercise also explored the dimension of the areas of focus of the published articles. The findings show that most studies (42%) were about the execution of the AI, ML, DL and IoT in clinical trials. The other major dimensions identified include technical details of the technologies (27%) and research (25%) regarding their usefulness in clinical trials. Policy (1%) and regulation (5%) received the least attention.

### Publication years

Analysis of the articles according to the years in which they were published gave an overview of the changes in the integration of AI, ML, DL, and IoT into clinical trials over the last 10 years. Research interest in this area has been consistently growing as shown by the publication of a third of the total articles in the years 2018, 2019, and 2020 (Fig. 7). Even though the mapping was done only two months into 2020, about 3% of the articles obtained were published in 2020. Thus, the increase in related publications for 2020 can be projected to reach about 18–20%, which will be an improvement from 15% and 16% published in 2018 and 2019 respectively. The proportions of articles published in 2015, 2016 and 2017 were relatively equal, averaging 11% of the total. Between 2010 and 2012, the average was 6%, which improved to 7.5% between 2013 and 2014. Therefore, the general trend is the increased focus on the utilization of AI, ML, DL, and IoTs in clinical trials.

## Discussion

### Principal findings

#### Evolution of research

A previous mapping review by Mehta et al. (2019) opened up discussions about the application of AI, ML, DL, and IoT in various fields within healthcare. By conducting a broad systematic mapping study covering all healthcare areas, the authors showed how big data analytics and AI are taking shape in improving various healthcare activities (Mehta et al. 2019). To the best of our knowledge, a systematic mapping study exploring the application of AI, ML, DL, and IoT in clinical trials is yet to be published. This systematic mapping study fills the gap by mapping all the studies that entail the application of advanced technologies to enhance recruitment, patient-matching, interventions, and monitoring of clinical trials.

**Fig. 4:**
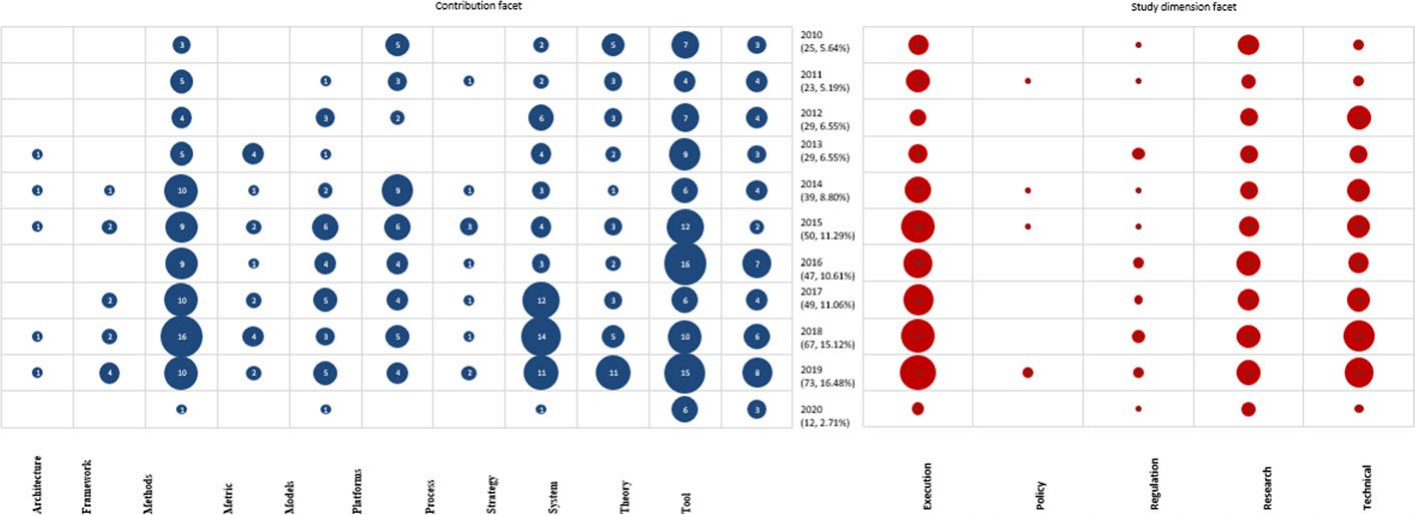
Bubble plot showing the trend of research according to contribution and study dimension, bubble size represents the number of articles

The mapping exercise to understand the trends of publications between January 2010 and February 2020 was completed by constructing correlational matrices visually presented as bubble plots. In Fig. 3, the 443 articles obtained during the search were categorized according to their research type and study focus and correlated with their year of publication. Similarly, Fig. 4 shows a distribution of the publications by their contribution and study dimension since 2010.

The maps reveal a trend of increasing integration of the AI, ML, DL, and IoT in clinical trials between 2010 and 2020. Evaluation studies almost doubled from 7 to 2010 to 13 in 2019. Validation studies quadrupled from 7 to 2010 to 27 in 2019. While there was only a single solution proposal in 2010, in 2018 they were 10, indicating a tenfold increase. The increased application of advanced technologies is also evident in the recent high rate of publication of experience papers. Out of all the 20 experience papers published between 2010 and 2020, 60% (12 out of 20) were published after 2018. A similar trend is evident in the review and discussion papers. The 2013–2015 period is characterized by the highest number of philosophical articles. Slightly more than 50% (13/24) of the philosophical papers were published in 2013, 2014 and 2015. Study protocols were common in 2017, 2018, and 2019. Therefore, there is a consistent pattern of improvement in the quality of the research types conducted in the field.

Analysis by study focus shows that they were about the application of the AI, ML, DL, and IoTs in conducting clinical trials generally as shown by the predominant overview studies. The specific focus area that received the most attention is recruitment, followed by eligibility. The implication is that advanced technologies could be most useful in the pre-trial stages of the RCTs. Patient matching, which is an aspect of recruitment, has received the same attention across the years, as shown by an average of 2 articles every year regarding it. Using the technologies in administering or monitoring interventions is also emerging as revealed by the publication of 80% (24/30) articles in the second half (2016–2020) of the study period (Fig. 3). The enhancement of adherence to ethical standards, more particularly consenting procedures, has also been receiving some interest since 2013. Therefore, beyond the pre-trial screening, AI, ML, DL, and IoT seem to be gaining tract in the conducting of the clinical trials.

The results of this mapping exercise also reveal an upward trend in the number of studies that entail the execution of clinical trials using advanced technologies. Since 2015, more than 20 studies per year entailed execution of the AI, ML, DL, and IoT in clinical trials as shown in the study dimension part of Fig. 4. The publications tripled from 10 to 2010 to 31 in 2019. Technical papers, which mainly inform the execution modalities, increased sevenfold from 3 to 2010 to 21 in 2019, thus indicating a rising intent to make the advanced technologies improve the implementation of clinical trial plans.

It is also evident that scientists have been keen on understanding the area of interest based on the publication of related research papers every year, 5–15 articles per year. Moreover, there could be intentions to have the AI, ML, DL, and IoT feature in clinical trial policies as shown by a publication of three articles in 2019, which constitute 50% (3/6) of the policy-related articles since 2010. The regulation dimension attracted 1–4 studies per year, implying little but persistent concern to have the improved clinical trials adhere to regulations.

#### Focus of research studies

**Fig. 5:**
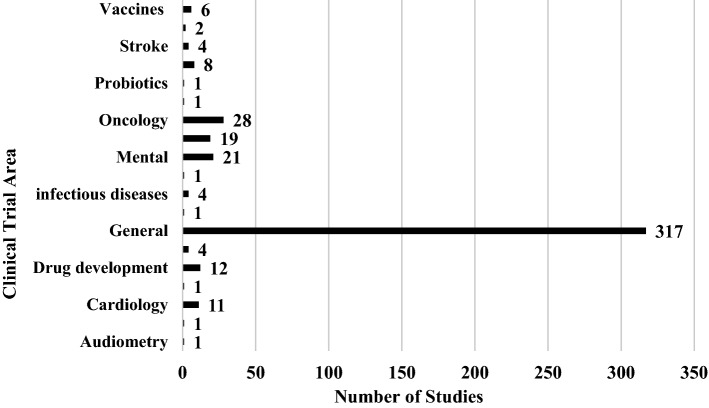
Advanced technologies by their categories and by their frequency of usage in studies

This mapping study indicates that the application of AI, ML, DL, and IoTs is happening in various clinical areas where clinical trials are conducted. However, more than 70% (317/443) studies were general in that they explored the application of the advanced technologies in clinical trials as a whole, without specifying the clinical area as shown in Fig. 5. The 30% (126/443) studies that focused on individual clinical areas comprise 6% (28/443) oncology trials, 5% (21/443) mental health trials, and 4% (19/443) clinical trials on metabolic disorders. Other common clinical areas include drug development (12), cardiology (11), respiratory diseases (8), and vaccine development (8).

**Fig. 6:**
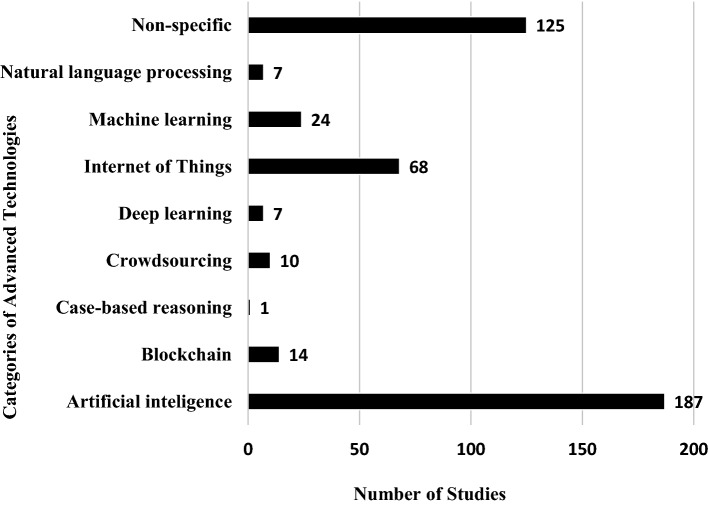
Distribution of research as per clinical trial areas

Regarding the type of advanced technology applied, most of the studies entailed the use of artificial intelligence (42%, 187/443) as shown in Fig. 6. More than a quarter of the studies (125/443) did not focus on any specific advanced technology; they handled them as a conglomerate. Fifteen percent (68/443) of the articles were about the application of IoT. ML and DL were applied in a few studies, 24 and 7 respectively. Blockchain (14), crowdsourcing (10), natural language processing (7), and case-based reasoning (1) were individually applied in some studies. The distribution on the technical front shows that artificial intelligence is the commonest advanced technology in clinical trials.

Regarding the contribution of the research studies, the current high presence of methods and theories implies a need to research other aspects. Architecture, processes, and metrics, which received little attention as shown in Table 7 in the appendix, are vital in informing the integration of the advanced technologies into clinical trials hence they should receive researchers’ attention.

### Comparison to related studies

The findings of this mapping study are consistent with the results of a research on the use of big data analytics and artificial intelligence in healthcare (Mehta et al. 2019), since both show a trend of increasing numbers of empirical studies, such as evaluation and validation studies, over the years. However, since the clinical trials area is a small part of healthcare, this mapping exercise only entailed 443 articles compared to the 2421 articles in the map covering the entire healthcare sector (Mehta et al. 2019). The focus on clinical trials only facilitated a thorough identification of the articles that use the advanced technologies in clinical trials.

**Fig. 7:**
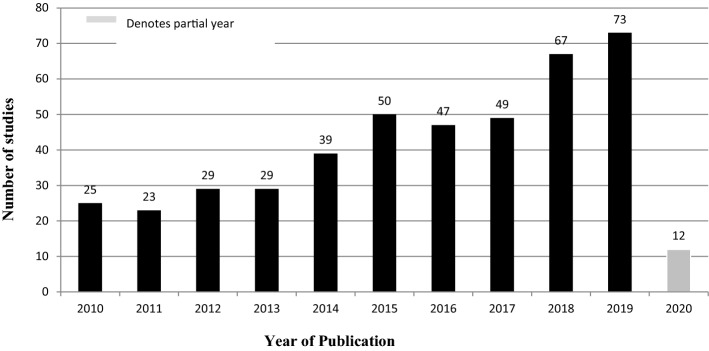
Publication distribution by year

The results displayed in Fig. 7 show a similar to the findings by Mehta et al. (2019). The number of relevant publications increased threefold from only 25 in 2010 to 73 in 2019. It means that in the future, more researchers will focus on enhancing clinical trials using advanced technologies. The clinical areas prominent in this study, oncology, is also one of the key areas in the article by Mehta et al. (2019). However, the integration of the advanced technologies in the general design and implementation of clinical trials, which is the focus of the current study, is scantly mentioned by Mehta et al. (2019), hence this study can serve to fill the gap left by the mapping exercise targeting the entire healthcare.

### Limitations of the study

Some of the possible limitations of this mapping study include selection bias and misclassification bias. The inclusion of the articles into the map and deciding where to categorize them was based on the judgment of the authors; there were no calibrated tools for the process. Hence, the risk of the authors’ inclinations affecting the selection and classification of the articles existed. It was minimized by involving all the authors in deciding on the selection of the articles and their placing into the various categories. Thus, consensus greatly mitigated this limitation.

Another limitation is the possible incompleteness of the identification of relevant articles. Although the search process was thorough, the risk of missing out some articles or inaccurately excluding them was a reality. The limitation was addressed by intensifying the search process through checking suggestions of articles related to the obtained articles and reexamining excluded articles to verify their unsuitability. Despite the measures, it is still possible that some relevant publications may have not been identified.

### Implications for future research and practice

This mapping study can serve as a snapshot of the status of the application of advanced technologies in clinical trials. Researchers planning to study or implement AI, ML, DL, or IoTs in clinical trials can get a picture of the current state of affairs in the field by reviewing this systematic map. Besides, the map makes it easy for interested researchers to identify the areas that have received little focus or the promising areas where they can invest their research efforts to enrich the field. This study also reveals to scientists that the current trend in research in this field necessitates conducting validation and evaluation studies, and publishing experience papers. Empirical research and real-life testing are taking center-stage in exploring the utility of AI, ML, DL, and IoT in clinical trials. The findings of the study also point to areas where clinical trial experts can partner with information technology specialists to enhance the utilization of advanced technologies to attain efficiency, timeliness, and cost-effectiveness in the planning and execution of clinical trials.

## Conclusion

This systematic map offers a view of the current state of research regarding AI, ML, DL, and IoTs in clinical trials. Its creation entailed identifying a total of 443 articles and classifying them to support the understanding of the field in the best way possible. The classification process was systematic; thus it can be reproduced. The study can be considered as a subset and an augment of a previous systematic map by Mehta, Pandit, and Shukla that included studies about big data analytics and AI in the entire healthcare field. It can serve as a reference base for research efforts whose aim is improving clinical trials through advanced technologies.

The study revealed that the number of publications in this area has been rising since 2010, and the studies are progressively embracing an empirical approach. By categorizing the studies according to their research types, study focus, contributions, and study dimension; and examining their changes in numbers annually from 2010 to 2020, research trends in the field emerged. The systematic map serves as a snapshot of the research status in the field and can serve as a base for identifying technical strategies and research gaps that can facilitate the refining of clinical trials using emerging technologies. The comprehensive overview described through the systematic mapping process in this study was scantly captured in previous reviews, thus the article provides new information. Overall, it is evident that advanced technologies are transforming the design and execution of clinical trials.

## Appendix

See Tables 6 and 7.

**Table 6:** Shows the research types, their proportions, and counts

Research type	Proportion of study type (%)	Count of study type
Discussion paper	12.87	57
Evaluation study	20.99	93
Experience paper	4.51	20
Opinion paper	0.90	4
Philosophical paper	5.42	24
Review paper	8.80	39
Solution proposal	16.48	73
Study protocol	2.03	9
Validation study	27.99	124
Grand Total	100.00	443

**Table 7:** Shows the research types under each category of contribution

Contribution and research type	Count of study type (%)	Count of study type
Architecture	**1.13**	**5**
Validation study	0.23	1
Philosophical paper	0.23	1
Solution proposal	0.68	3
Framework	**2.48**	**11**
Validation study	0.68	3
Evaluation study	0.45	2
Review paper	0.23	1
Discussion paper	0.68	3
Solution proposal	0.45	2
Methods	**18.51**	**82**
Validation study	7.45	33
Evaluation study	4.06	18
Review paper	0.23	1
Discussion paper	1.35	6
Philosophical paper	0.23	1
Experience paper	1.13	5
Solution proposal	3.16	14
Study protocol	0.68	3
Opinion paper	0.23	1
Metric	**3.61**	**16**
Validation study	2.26	10
Evaluation study	0.90	4
Philosophical paper	0.23	1
Solution proposal	0.23	1
Models	**7.00**	**31**
Validation study	2.48	11
Evaluation study	1.35	6
Review paper	0.90	4
Discussion paper	0.23	1
Solution proposal	1.58	7
Study protocol	0.45	2
Platforms	**9.48**	**42**
Validation study	2.26	10
Evaluation study	2.93	13
Review paper	0.90	4
Discussion paper	0.45	2
Experience paper	1.13	5
Solution proposal	1.81	8
Process	**2.26**	**10**
Validation study	1.13	5
Evaluation study	0.90	4
Experience paper	0.23	1
Strategy	**14.00**	**62**
Validation study	3.61	16
Evaluation study	3.61	16
Review paper	0.68	3
Discussion paper	1.13	5
Philosophical paper	0.68	3
Experience paper	0.90	4
Solution proposal	2.93	13
Study protocol	0.45	2
System	**8.58**	**38**
Validation study	3.16	14
Evaluation study	2.03	9
Review paper	0.23	1
Discussion paper	1.13	5
Philosophical paper	0.45	2
Experience paper	0.23	1
Solution proposal	1.35	6
Theory	**22.12**	**98**
Validation study	0.23	1
Evaluation study	0.90	4
Review paper	5.64	25
Discussion paper	7.67	34
Philosophical paper	3.61	16
Experience paper	0.23	1
Solution proposal	2.93	13
Study protocol	0.23	1
Opinion paper	0.68	3
Tool	**10.84**	**48**
Validation study	4.51	20
Evaluation study	3.84	17
Discussion paper	0.23	1
Experience paper	0.68	3
Solution proposal	1.35	6
Study protocol	0.23	1
Grand total	**100.00**	**443**

Bold shows the categorization of the studies in consideration to their contributions
